# Identification of enzymes responsible for extracellular alginate depolymerization and alginate metabolism in *Vibrio algivorus*

**DOI:** 10.1007/s00253-016-8021-7

**Published:** 2016-12-03

**Authors:** Hidetaka Doi, Yuriko Tokura, Yukiko Mori, Kenichi Mori, Yoko Asakura, Yoshihiro Usuda, Hiroo Fukuda, Akito Chinen

**Affiliations:** 1 Process Development Laboratories, Research Institute for Bioscience Products & Fine Chemicals, Ajinomoto Co., Inc., 1-1 Suzuki-cho, Kawasaki-ku, Kawasaki-shi, Kanagawa 210-8681 Japan; 2 Department of Biological Sciences, Graduate School of Science, The University of Tokyo, 7-3-1 Hongo, Bunkyo-ku, Tokyo, 113-0033 Japan; 3 Frontier Research Laboratories, Institute for Innovation, Ajinomoto Co., Inc., 1-1 Suzuki-cho, Kawasaki-ku, Kawasaki-shi, Kanagawa 210-8681 Japan

**Keywords:** Alginate, *Vibrio algivorus*, Alginate lyase, Polysaccharide-degrading enzyme, Alginate metabolism, Biorefinery

## Abstract

Alginate is a marine non-food-competing polysaccharide that has potential applications in biorefinery. Owing to its large size (molecular weight >300,000 Da), alginate cannot pass through the bacterial cell membrane. Therefore, bacteria that utilize alginate are presumed to have an enzyme that degrades extracellular alginate. Recently, *Vibrio algivorus* sp. SA2^T^ was identified as a novel alginate-decomposing and alginate-utilizing species. However, little is known about the mechanism of alginate degradation and metabolism in this species. To address this issue, we screened the *V. algivorus* genomic DNA library for genes encoding polysaccharide-decomposing enzymes using a novel double-layer plate screening method and identified *alyB* as a candidate. Most identified alginate-decomposing enzymes (i.e., alginate lyases) must be concentrated and purified before extracellular alginate depolymerization. AlyB of *V. algivorus* heterologously expressed in *Escherichia coli* depolymerized extracellular alginate without requiring concentration or purification. We found seven homologues in the *V. algivorus* genome (*alyB*, *alyD*, *oalA*, *oalB*, *oalC*, *dehR*, and *toaA*) that are thought to encode enzymes responsible for alginate transport and metabolism. Introducing these genes into *E. coli* enabled the cells to assimilate soluble alginate depolymerized by *V. algivorus* AlyB as the sole carbon source. The alginate was bioconverted into l-lysine (43.3 mg/l) in *E. coli* strain AJIK01. These findings demonstrate a simple and novel screening method for identifying polysaccharide-degrading enzymes in bacteria and provide a simple alginate biocatalyst and fermentation system with potential applications in industrial biorefinery.

**Electronic supplementary material:**

The online version of this article (doi:10.1007/s00253-016-8021-7) contains supplementary material, which is available to authorized users.

## Introduction

Alginate is an abundant sugar in marine brown macroalgae (Chapman 1970) that is considered as an efficient and non-food-competing candidate raw material for biorefinery. Sugars from cane or corn starch are currently the major raw materials of biorefinery; however, an ethical challenge associated with their production is that it competes with food production. In contrast, the cultivation of marine brown macro algae does not require arable land, fresh water, pesticide, or fertilizer (John et al. 2011) and has the advantage of rapid growth (Stephens et al. 2013).

Alginate consists of a long-chain polymer of α-l-guluronic acid and β-d-mannuronic acid that form a high molecular weight macromolecule (>300,000 Da) that is poorly soluble in water (Gacesa 1988). Raw alginate is too large to import through the cell membrane. As such, most microorganisms cannot degrade and utilize alginate. However, novel alginate-utilizing microbial species have recently been discovered (Kita et al. 2015; Doi et al. 2016), while various fermentation processes using alginate as raw material have been proposed. For example, ethanol for biofuel production has been derived from alginate by fermentation using metabolically engineered *Sphingomonas* sp. A1 strain (Takeda et al. 2011), *Escherichia coli* (Wargacki et al. 2012), and *Saccharomyces cerevisiae* (Enquist-Newman et al. 2014), while pyruvate has been produced by *Sphingomonas* sp. A1 (Kawai et al. 2014). These studies exploit specific alginate-assimilating species and/or their enzymes. However, there are certain challenges for the industrialization of alginate fermentation, including the need to pre-treat alginate for degradation and ensuring efficient bioconversion of alginate into products. These can potentially be circumvented by identifying novel alginate-degrading and alginate-utilizing enzymes, which could be used for the production of commodity chemicals such as l-lysine, a food and feed additive. To date, there have been no reports of l-lysine production from alginate as a carbon source, although the bioconversion of d-glucose to l-lysine in *E. coli* strain AJIK01 has been described (Doi et al. 2015).

We recently isolated *Vibrio algivorus* sp. strain SA2^T^ from the gut flora of the turban shell marine snail (Doi et al. 2016), which can depolymerize and assimilate alginate as a sole carbon source, although the underlying mechanisms are unclear. To address this issue, in the present study we developed a novel system to screen for genes encoding extracellular active alginate-degrading enzymes and identified *V. algivorus alyB* as a candidate*.* The protein was expressed in *E. coli* and exhibited extracellular alginate-depolymerizing activity. We also identified seven putative alginate utilization pathway genes in *V. algivorus* (*toaA*, *alyB*, *alyD*, *oalA*, *oalB*, *oalC*, and *dehR*) that were expressed in wild-type *E. coli* and conferred the cells with the capacity to convert depolymerized alginate into l-lysine. This is the first report identifying genes encoding enzymes for alginate degradation and utilization in *V. algivorus* and demonstrating the bioconversion of alginate into l-lysine.

## Materials and methods

### Bacterial strains and plasmids

All strains and plasmids used in this study are listed in Table 1. Primers used for the construction of plasmids and strains are listed in Table S1. DNA fragments were PCR-amplified and purified with the Wizard SV Gel and PCR Clean-up system (Promega, Madison, WI, USA). To construct plasmids, purified insert DNA was cloned into the linearized vector with the In-Fusion HD PCR cloning system (Clontech, Mountain View, CA, USA). To express *V. algivorus* sp. SA2^T^ genes (*dehR*, *alyB*, *alyD*, *oalA*, *oalB*, and *oalC*) in *E. coli*, the genes were PCR amplified and inserted downstream of the chloramphenicol resistance marker gene *cat* and the promoter sequence attR-cat-attL-P_14_ using the plasmids shown in Table 1 as a template (i.e. pM08, pM03, pM09, pM10, pM11, and pM12) and the λ-red system (Datsenko and Wanner 2000). The *cat* gene was excised from the genome as previously described (Katashkina et al. 2009). To express the *V. algivorus* sp. SA2^T^ *toaA* gene in *E. coli*, we carried out crossover PCR amplification of *toaA* (using *V. algivorus* sp. SA2^T^ genomic DNA as the template) and inserted the amplicon downstream of attR-cat-attL-P_tac1000_, which was amplified using a chemically synthesized DNA template (Katashkina et al. 2005) and the λ-red system. The *cat* gene was then excised from the genome as described above.

**Table 1:** Strains and plasmids

Strain	Description or genotype	Reference
MG1655	*Escherichia coli*, F^−^λ^−^ *ilvG rfb-50 rph-1*	CGSC (no. 6300)
*Vibrio algivorus* SA2^T^	Alginate-utilizing strain	DSM 29824^T^; Doi et al. 2016
*Vibrio splendidus* ATCC33125^T^	Alginate-utilizing strain	Le Roux et al. 2009
JM109	*E. coli*, *recA1*, *endA1*, *gyrA96*, *thi*-*1*, *hsdR17*(*r*_*K*_^−^ *m*_*K*_^+^), *e14* ^−^ (*mcrA* ^−^), *supE44*, *relA1*, *Δ*(*lac-proAB*)/F′*traD36*, *proAB* ^+^, *lac I* ^*q*^, *lacZΔ*M15	Takara Bio, Kyoto, Japan
EPI300	*E. coli*, F^−^ *mcrAΔ*(*mrr-hsdRMS*-*mcrBC*) *Φ80dlacZΔ*M15*ΔrecA1 endA1 araD139 lacX74Δ*(*ara, leu*)*7697 galU galK* λ^−^ *rpsL nupG trfA tonA dhfr*	Epicentre Biotechnologies, Madison, WI, USA
D2964	MG1655*ΔnarI*::P_14_-*dehR*, *ΔycgV*::P_14_-*alyB*, *Δ*ycgG::P_14_-*alyD*, *ΔyegQ*::P_14_-*oalA*, *ΔybdN*::P_14_-*oalB*, *ΔyggW*::P_14_-*oalC*	This study
D2978	MG1655*ΔnarI*::P_14_-*dehR*, *ΔycgV*::P_14_-*alyB*, *Δ*ycgG::P_14_-*alyD*, *ΔyegQ*::P_14_-*oalA*, *ΔybdN*::P_14_-*oalB*, *ΔyggW*::P_14_-*oalC*, *ΔyegD*::P_tac6_-*toaA*	This study
AJIK01	*E. coli* strain capable of l-lysine bioconversion	Doi et al. 2015; NITE-BP1520
D3000	AJIK01*ΔnarI*::P_14_-*dehR*, *ΔycgV*::P_14_-*alyB*, *Δ*ycgG::P_14_-*alyD*, *ΔyegQ*::P_14_-*oalA*, *ΔybdN*::P_14_-*oalB*, *ΔyggW*::P_14_-*oalC*, *ΔyegD*::P_tac6_-*toaA*	This study
pCC1FOS	Fosmid vector for preparing the *V. algivorus* genomic library	Epicentre Biotechnologies
pM01	Plasmid for cloning and serving as a vector control, pMW119-attR-*cat*-attL-P_14_	Doi et al. 2015
pM02	Plasmid expressing *alyB* of *V. splendidus*, pMW119-attR-*cat*-attL-P_14_-*alyB*	This study
pM03	Plasmid expressing *alyB* of *V. algivorus*, pMW119-attR-*cat*-attL-P_14_-*alyB*	This study
pM04	Plasmid expressing SP-deficient *alyB* mutant of *V. algivorus*, pMW119-attR-*cat*-attL-P_14_-*alyBΔ*SP	This study
pM05	Plasmid expressing CBM32-deficient *alyB* mutant of *V. algivorus*, pMW119-attR-*cat*-attL-P_14_-*alyBΔ*CBM32	This study
pM06	Plasmid expressing PL7-deficient *alyB* mutant of *V. algivorus*, pMW119-attR-*cat*-attL-P_14_-*alyBΔ*PL7	This study
pM07	Plasmid expressing SP- and CBM32-deficient *alyB* mutant of *V. algivorus*, pMW119-attR-*cat*-attL-P_14_-*alyBΔ*SP*Δ*CBM32	This study
pM08	Plasmid expressing *dehR* of *V. algivorus*, pMW119-attR-*cat*-attL-P_14_-*dehR*	This study
pM09	Plasmid expressing *alyD* of *V. algivorus*, pMW119-attR-*cat*-attL-P_14_-*alyD*	This study
pM10	Plasmid expressing *oalA* of *V. algivorus*, pMW119-attR-*cat*-attL-P_14_-*oalA*	This study
pM11	*oalB* of *Vibrio algivorus* expressing plasmid,pMW119-attR-*cat*-attL-P_14_-*oalB*	This study
pM12	*oalC* of *Vibrio algivorus* expressing plasmid,pMW119-attR-*cat*-attL-P_14_-*oalC*	This study
pKD46	λ-Red system helper plasmid	Datsenko and Wanner 2000
pMW-intxis-ts	λ-Red system marker excision plasmid, temperature sensitive	Katashkina et al. 2009
pMW118-attR-cat-attL-P _tac6_	Template plasmid for cloning of attR-cat-attL-P _tac6_-*toaA* by crossover PCR	Katashkina et al. 2005

*CGSC* Coli Genetic Stock Center

### Screen for extracellular active alginate lyase

We prepared sheared 40–50 kb DNA fragments of *V. algivorus* sp. SA2^T^ genomic DNA using HydroShear (Gene Machines, San Carlos, CA, USA) followed by gel purification. *E. coli* EPI300 cells were transformed with the sheared fragments as previously described (Wargacki et al. 2012). *E. coli* EPI300 colonies expressing the fragments were covered with M9 minimal medium (Miller 1992) containing 4 g/l agar and 10 g/l sodium alginate (300–400 cP, CAS no. 9005-38-3; Wako Pure Chemical Industries, Osaka, Japan) (Fig. S1A). After a 16-h incubation at 37 °C, we observed an indentation over the colonies (Fig. S1B), which were transferred to a Luria-Bertani (LB) plate containing 12.5 mg/l chloramphenicol (Cm), 1 mM isopropyl β-d-1-thiogalactopyranoside, and 40 μg/l X-gal. Colonies were then cultured in liquid LB medium containing 12.5 mg/l Cm at 37 °C with constant shaking at 120 rpm until the optical density at 600 nm (OD_600_) was 0.8. We added 0.1% (*v*/*v*) of Copy Control Induction Solution (Epicentre, Madison, WI, USA) and incubated the cultures at 37 °C for 6 h with constant shaking at 120 rpm. Cells were collected by centrifugation for 20 min at 3000×*g* and fosmid DNA was extracted using the Plasmid Midi kit (Qiagen, Hilden, Germany). The terminal sequences of extracted fosmids were determined by Sanger sequencing using EPI forward and reverse primers (Table S1). The whole insert sequence of the extracted fosmid was determined based on the draft genome of SA2^T^ (Doi et al. 2016).

### In vitro determination of alginate lyase activity

*V. splendidus* ATCC33125^T^ and *V. algivorus* sp. SA2^T^ cells were grown on LB plates with 15 g/l NaCl for 16 h at 30 °C. The cells were scraped and crude cell lysates were obtained using BugBuster Master Mix (Merck Millipore, Billerica, MA, USA). A similar procedure was used to obtain crude cell lysates of *E. coli* grown on LB plates with 40 mg/l Cm for 16 h at 30 °C. Protein concentration of the lysates was measured as previously described (Chial and Splittgerber 1993) using Coomassie Brilliant Blue (CBB) G (Nacalai Tesque, Kyoto, Japan). Alginate lyase activity was measured using a published protocol (Iwamoto et al. 2001; Tang et al. 2009). Briefly, 180 μl of the reaction mixture (0.08 g/l protein sample, M9 minimal medium, and 2 g/l sodium alginate) was transferred to a 96-well microplate (Greiner Bio One, Frickenhausen, Germany) followed by incubation at different temperatures (34, 37, 40, and 44 °C). After 18 min, the increase in absorbance at 235 nm (Abs_235_) was measured with a Spectra Max190 microplate reader (Molecular Devices, Sunnyvale, CA, USA). One unit of alginate lyase activity was defined as an increase in Abs_235_ of 0.100 per minute (Iwamoto et al. 2001).

### Alginate viscosity test for detecting in vivo extracellular alginate-decomposing activity

*E. coli* cells were cultured in 5 ml of LB with 40 mg/l Cm at 37 °C for 16 h with shaking at 120 rpm. Sodium alginate (0.25 g) was added to the test tubes—which were placed at an angle of 45°—and the cultures were incubated with shaking for 72 h. We waited for 5 s after placing the tubes upright to assess the angle of the liquid surface (Fig. 2). If alginate depolymerization was insufficient, the liquid surface remained at an angle of 45 (Fig. 2a); conversely, upon alginate depolymerization, the liquid loses its viscosity and the liquid surface would be horizontal (Fig. 2b). We analysed the depolymerized liquid by gel permeation chromatography (GPC) and confirmed that the alginate peak was reduced (Fig. 3b and data not shown for the other results in Table 2).

**Table 2:** Results of the alginate viscosity test for detecting in vivo extracellular alginate-decomposing activity

	1	2	3	4	5	6	7
Whole broth	−	−	+	−	−	+	−
Supernatant	−	−	+	−	−	+	−
Washed cells	−	−	+	−	−	+	−
Cell lysate	−	+	+	+	−	+	+

1, JM109/pM01 (vector control); 2, JM109/pM02 (expressing wild-type AlyB of *V. splendidus*); 3, JM109/pM03 (expressing wild-type AlyB of *V. algivorus*); 4, JM109/pM04 (SP deletion mutant); 5, JM109/pM06 (PL7 deletion mutant); 6, JM109/pM05 (CBM32 deletion mutant); 7, JM109/pM07 (SP and CBM32 deletion mutant)

### Preparation of supernatant and washed cell samples

*E. coli* strains were grown overnight at 37 °C on LB plates. Cells were then inoculated into 40 ml of fermentation medium composed of 20 g/l glucose, 5 g/l tryptone, 2.5 g/l yeast extract, 5 g/l NaCl, 40 mg/l Cm, and 0.3 M 3-(N-morpholino) propanesulphonic acid (MOPS; adjusted to pH 7.0 with NaOH) in a Sakaguchi flask at an initial OD_600_ of 0.05 at 37 °C for 20 h with shaking at 120 rpm. The cultures were centrifuged at 5000 rpm (7830×*g*) and 4 °C for 10 min (CR20GIII; Hitachi, Tokyo, Japan). The pellet was washed three times with 5 ml of 0.85% NaCl followed by centrifugation at 5000 rpm and 4 °C for 10 min.

### Determination of protein concentration in the supernatant

The supernatant from the above-described cultures was concentrated using the Amicon Ultra-15 centrifugal filter unit with an Ultracel-50 membrane (Merck Millipore). Protein concentration was measured with the CBB assay using bovine serum albumin (Bio-Rad, Hercules, CA, USA) as a standard.

### Preparation of alginate depolymerized with commercial alginate lyase

Sodium alginate (2.5 g) and 50 ml distilled water were mixed in a Sakaguchi flask (500 ml) at 120 rpm and 37 °C for 16 h to obtain a uniformly dispersed, clear alginate gel containing 50 g/l sodium alginate. We added 40 ml of 0.85% NaCl and 1 mg/l commercial alginate lyase (A1603; Sigma-Aldrich, St. Louis, MO, USA) with shaking at 120 rpm and 37 °C for 25 h. Depolymerized alginate solution was sterilized using Nalgene Rapid-flow filters (pore size 0.2 μm; Thermo Fisher Scientific, Waltham, MA, USA).

### Preparation of alginate depolymerized with alginate lyase purified from *E. coli*

*E. coli* strain JM109/pM03 was grown at 37 °C on LB plates containing 40 mg/l Cm for 16 h. Colonies were inoculated into 40 ml of fermentation medium composed of 20 g/l glucose, 5 g/l tryptone, 2.5 g/l yeast extract, 5 g/l NaCl, 40 mg/l Cm, and 0.3 M MOPS (adjusted to pH 7.0 with NaOH) in a Sakaguchi flask at an initial OD_600_ of 0.05 and incubated at 37 °C for 20 h with shaking at 120 rpm. Cells were collected by centrifugation at 5000 rpm and 4 °C for 10 min then inoculated in 0.85% NaCl such that the total volume was 40 ml. The mixture was combined with the uniformly dispersed clear alginate gel containing 50 g/l sodium alginate and incubated at 37 °C for 25 h with shaking at 120 rpm. After centrifugation at 5000 rpm and 4 °C for 10 min, the supernatant containing depolymerized alginate was recovered and sterilized using Nalgene Rapid-Flow filters (pore size 0.2 μm; Thermo Fisher Scientific) before addition of minimal medium.

### Test-tube cultivation with minimal medium

For test-tube cultivation on minimal medium, *E. coli* MG1655 and its derivative strains were grown overnight at 37 °C on LB plates. Colonies were inoculated into 5 ml M9 minimal medium supplemented with 1 mM MgSO_4_, 0.001% thiamine, and different carbon sources in L-shaped test tubes at an initial OD_600_ of 0.05 and incubated at 37 °C for 96 h with constant shaking at 70 rpm on a TVS062 CA rocking incubator (Advantec, Tokyo, Japan).

### Test-tube cultivation for l-lysine bioconversion

Test-tube cultivation for L-lysine bioconversion was carried out by growing *E. coli* AJIK01 and its derivative strains overnight at 37 °C on LB plates. Colonies were inoculated into 5 ml of medium composed of 0.25 g/l yeast extract, 4 g/l (NH_4_)_2_SO_4_, 0.01 g/l FeSO_4_∙7H_2_O, 0.01 g/l MnSO_4_·7H_2_O, 0.246 g/l MgSO_4_·7H_2_O, 0.001% thiamine, 10 g/l piperazine-*N*, *N*′-bis(2-ethanesulphonic acid) (adjusted to pH 7.0 with NaOH), and different carbon sources in test tubes at an initial dry cell weight of 0.05 g/l. Cells were cultured at 34 °C for 90 h with constant shaking at 120 rpm.

### Analytical procedures

Molecular weights and amounts of commercial alginate were determined by GPC under the conditions described in Table S2. Pullulan (CAS no. 9057-02-7) was used as the molecular weight standard. OD_600_ was measured with a U-2900 spectrophotometer (Hitachi). The standard error of the mean was calculated and a two-tailed unpaired Student’s *t* test was carried out using Excel software (Microsoft Corporation, Redmond, WA, USA) from more than three independent samples.

### Western blotting

Affinity-purified rabbit polyclonal antibody recognizing a chemically synthesized peptide sequence of *V. algivorus* AlyB (AAQKEARKDLRK) (Eurofins Genomics, Tokyo, Japan) was prepared as previously described (Iwai et al. 2015). Rabbit polyclonal anti-AlyB antibody (1:400) and horseradish peroxidase-linked anti-rabbit IgG (3:2000; Cell Signaling Technologies, Danvers, MA, USA; catalogue no. 7074) were used to detect AlyB. Two independent repeats were carried out for western blot analyses, for which 2 μg of each sample was used. Sodium dodecyl sulphate polyacrylamide gel electrophoresis was carried out using XV Pantera pre-cast gels (DRC Co., Tokyo, Japan) and SimplyBlue SafeStain solution (Thermo Fisher Scientific).

## Results

### Screen for extracellular alginate-decomposing enzyme in *V. algivorus* sp. SA2^T^ genome and phenotypic analysis of mutant AlyB

**Fig. 1:**
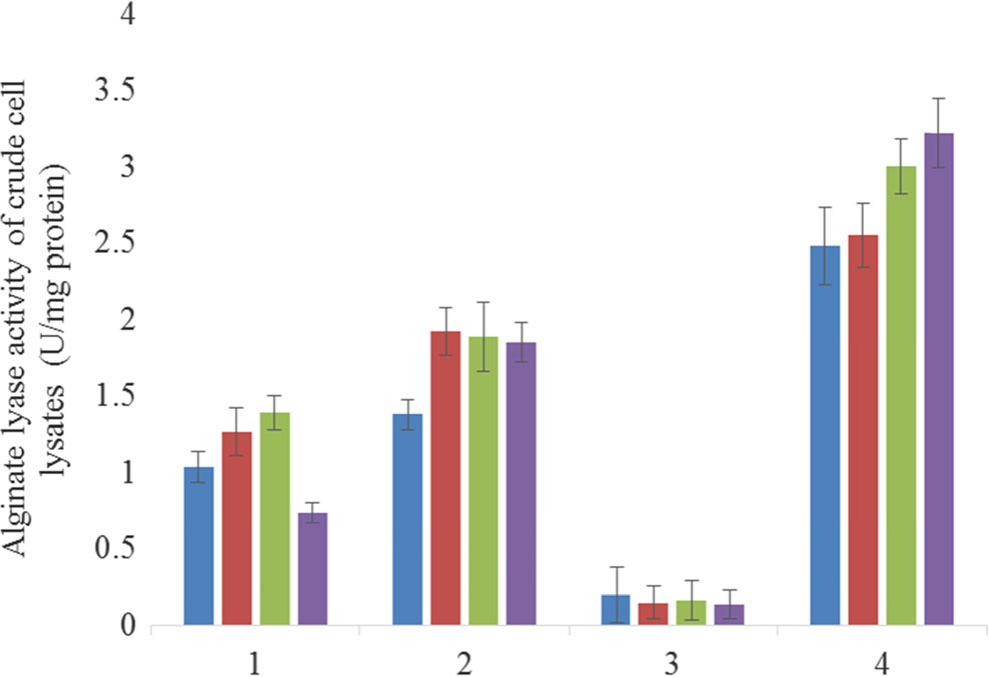
Results of alginate lyase enzyme activity assay. *1*, crude lysate of *V. splendidus* ATCC33125^T^; *2*, crude lysate of *V. algivorus* SA2^T^; *3*, crude lysate of *E. coli* JM109/pM01 (vector control); *4*, crude lysate of *E. coli* JM109/pM03 (harbouring *alyB* of *V. algivorus*). *Blue*, *red*, *green*, and *purple bars* represent activity at 34, 37, 40, and 44 °C, respectively

**Fig. 2:**
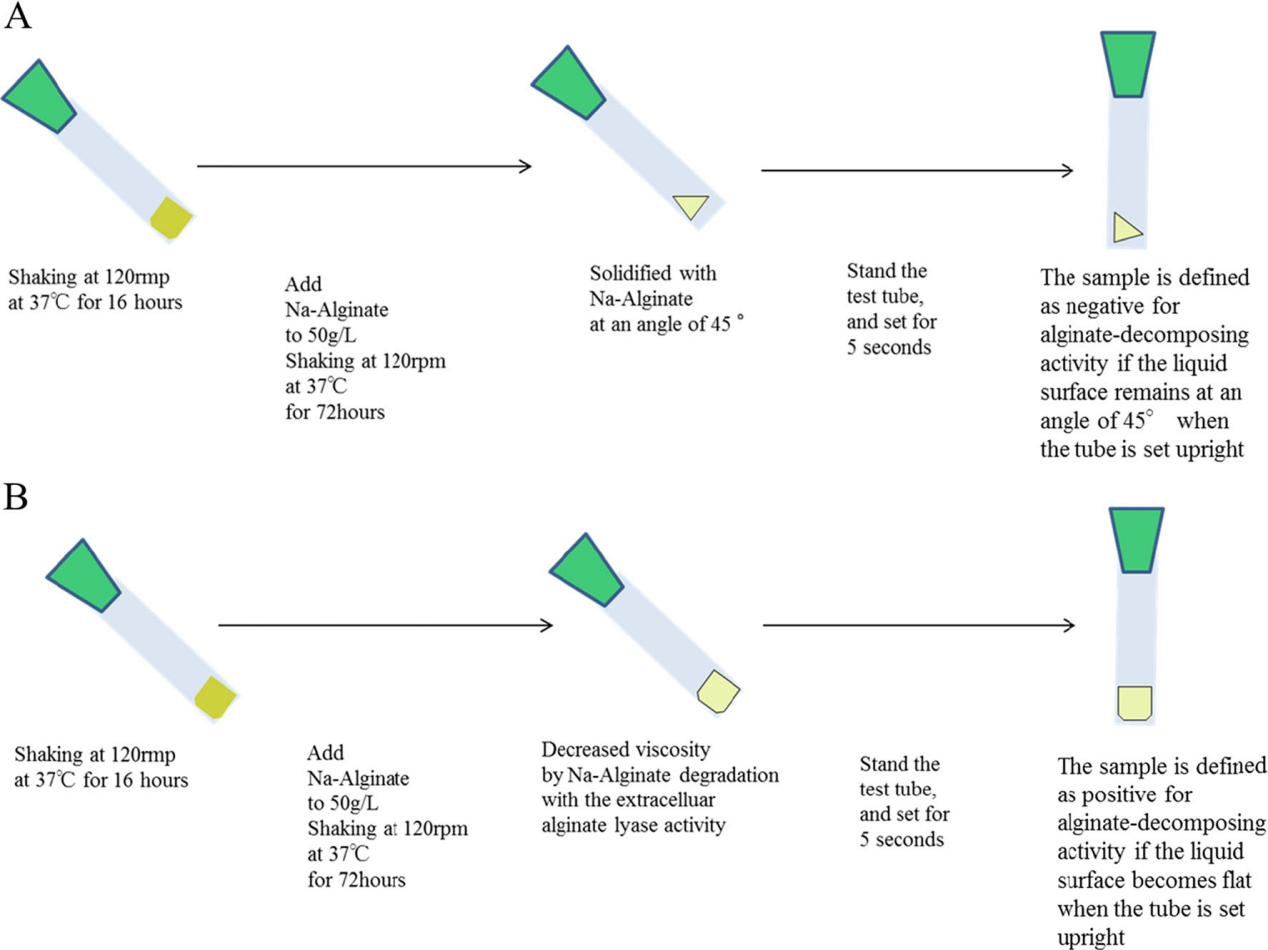
Schematic representation of the alginate viscosity test for detecting the in vivo extracellular alginate-decomposing activity. Schematic representation of negative (**a**) and positive (**b**) results

We used a plate assay method to screen colonies with extracellular alginate-decomposing activity (Fig. S1A, B). A colony of the SA2^T^ strain (Doi et al. 2016) was first prepared and covered with a layer of alginate-containing gel. A visible indentation in the gel formed over the colony (data not shown), which was presumed to result from the decomposition of alginate by the underlying colony. Over 3000 *E. coli* EPI300 colonies harbouring a fosmid containing a 40- to 50-kb fragment of SA2^T^ genomic DNA were inoculated on LB plates and covered with a layer of gel. After 16 h, three indentations appeared; the corresponding colonies were harvested and the fosmid DNA was extracted and sequenced. All three colonies harboured the same polysaccharide lyase family gene that showed high similarity to *alyB* of *V. splendidus* (Badur et al. 2015); we therefore named the gene *alyB* of *V. algivorus*. The gene was inserted into an expression plasmid (pM03) that was introduced into *E. coli* JM109 (Table 1). The in vitro alginate lyase activity of *V. algivorus* SA2^T^, *V. splendidus*, and *E. coli* JM109/pM03 whole cell lysates was analysed. *V. algivorus* SA2^T^ and *V. splendidus* lysates showed alginate lyase activities; the activities in the JM109/pM03 lysate were significantly higher than those in the *V. algivorus* SA2^T^ and *V. splendidus* lysates (Fig. 1). We also constructed a vector control strain (*E. coli* JM109/pM01) and a strain expressing *alyB* of *V. splendidus* (*E. coli* JM109/pM02) (Table 1). JM109/pM03 but not JM109/pM01 and JM109/pM02 grown on LB medium lowered the viscosity of alginate in the alginate viscosity test (Figs. 2a and S1).

**Fig. 3:**
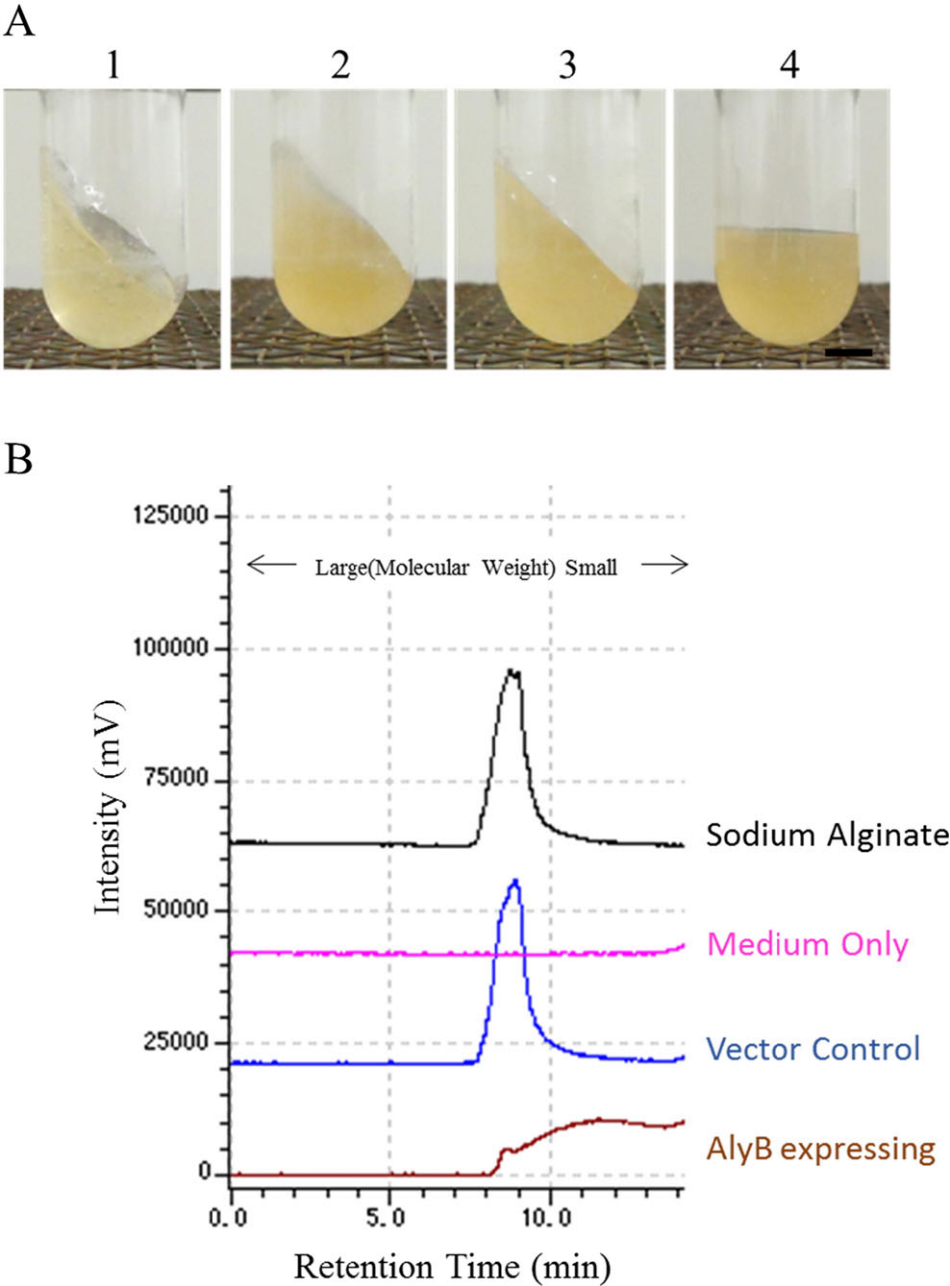
Extracellular alginate depolymerization by AlyB of *V. algivorus*. **a** Liquefaction of alginate-containing medium. *1*, LB medium with 50 g/l sodium alginate; *2*, JM109/pM01 broth with 50 g/l sodium alginate (vector control); *3*, JM109/pM02 broth with 50 g/l sodium alginate (whole broth of cells expressing *alyB* of *V. splendidus*); *4*, JM109/pM03 broth with 50 g/l sodium alginate (whole broth of cells expressing *alyB* of *V. algivorus*). **b** Results of GPC analysis. *Black*, 2 g/l sodium alginate standard; *pink*, LB medium; *blue*, 2 g/l sodium alginate after processing with the supernatant of JM109/pM01; *brown*, 2 g/l sodium alginate after processing with the supernatant of JM109/pM03

Alginate was added to the supernatants of JM109/pM01 and JM109/pM03 cultures, followed by incubation; the supernatants were then analysed by GPC (Fig. 3b). Alginate exhibited a single peak within the retention time of 7.7–10 min; the centre of the peak was at 8.7 min (Fig. 3b). The alginate peak was lower for the supernatant of JM109/pM03, although the peak was retained in the supernatant of JM109/pM01 (Fig. 3b).

**Fig. 4:**
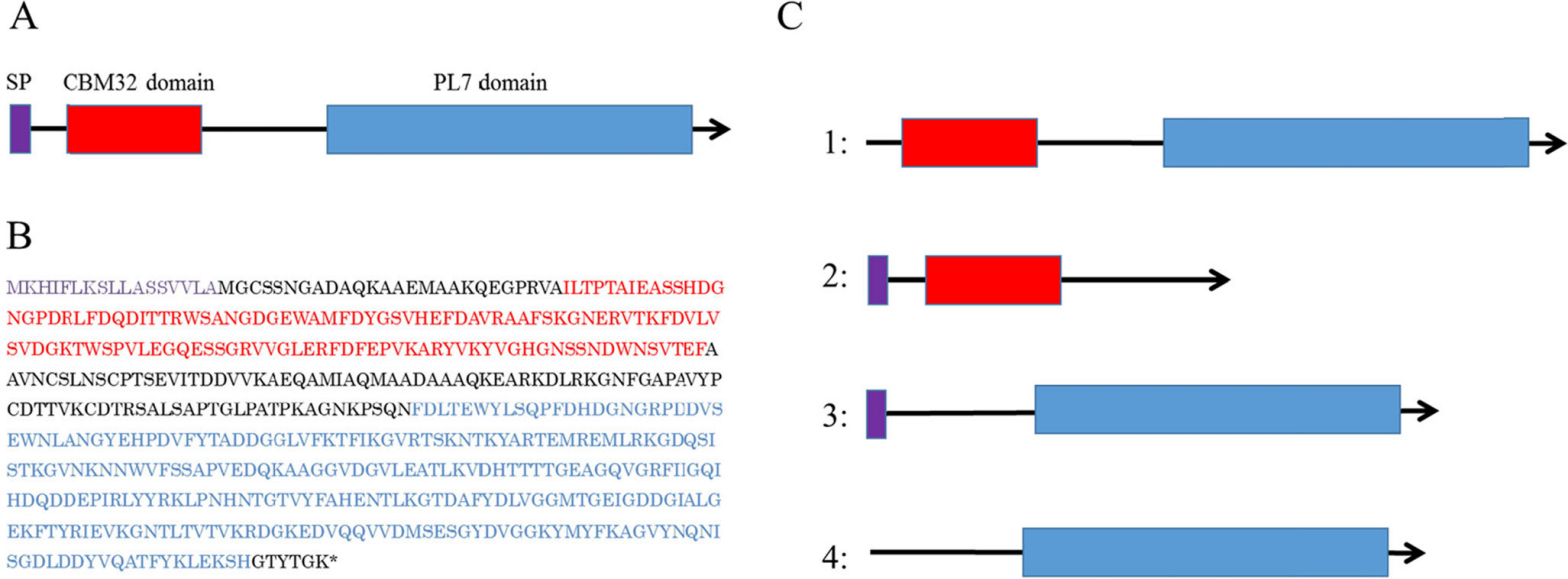
Predicted domain structure of *V. algivorus* AlyB. **a** The model was established using SignalP 4.0 (http://www.cbs.dtu.dk/services/SignalP/) (Petersen et al. 2011) and Pfam 25.0 (http://pfam.xfam.org) (Finn et al. 2014) software. **b** Amino acid sequence of *V. algivorus* AlyB. *Purple*, *red*, and *blue* letters denote the SP, CBM32, and PL7 domains, respectively. **c** Model of AlyB domain deletion mutants. *1*, SP deletion; *2*, PL7 deletion; *3*, CBM32 deletion; *4*, SP and CBM32 deletion

The domain structure of *V. algivorus* AlyB was modelled with SignalP 4.0 (http://www.cbs.dtu.dk/services/SignalP/; Petersen et al. 2011) and Pfam 25.0 (http://pfam.xfam.org; Finn et al. 2014) software. AlyB had a 17-amino acid (a.a.) signal peptide (SP) for secretion, a 123-a.a. CBM32 domain, and a 265-a.a. PL7 domain (Fig. 4a, b). We constructed plasmids harbouring AlyB sequences lacking each of these domains (Fig. 4c); the plasmids were then introduced into *E. coli* JM109 and the alginate viscosity test was performed (Fig. 2). The viscosity-reducing activity was lost in the supernatant of cells expressing *alyB* lacking the N-terminal SP (Table 2), while the cell lysate of the SP deletion mutant retained this activity. PL7 deletion also caused the loss of viscosity-lowering activity in the supernatant, washed cells, cell lysate, and whole broth (Table 2). Deletion of the CBM32 domain had no effect on viscosity, which was similar to that observed with wild-type *alyB* expression.

**Fig. 5:**
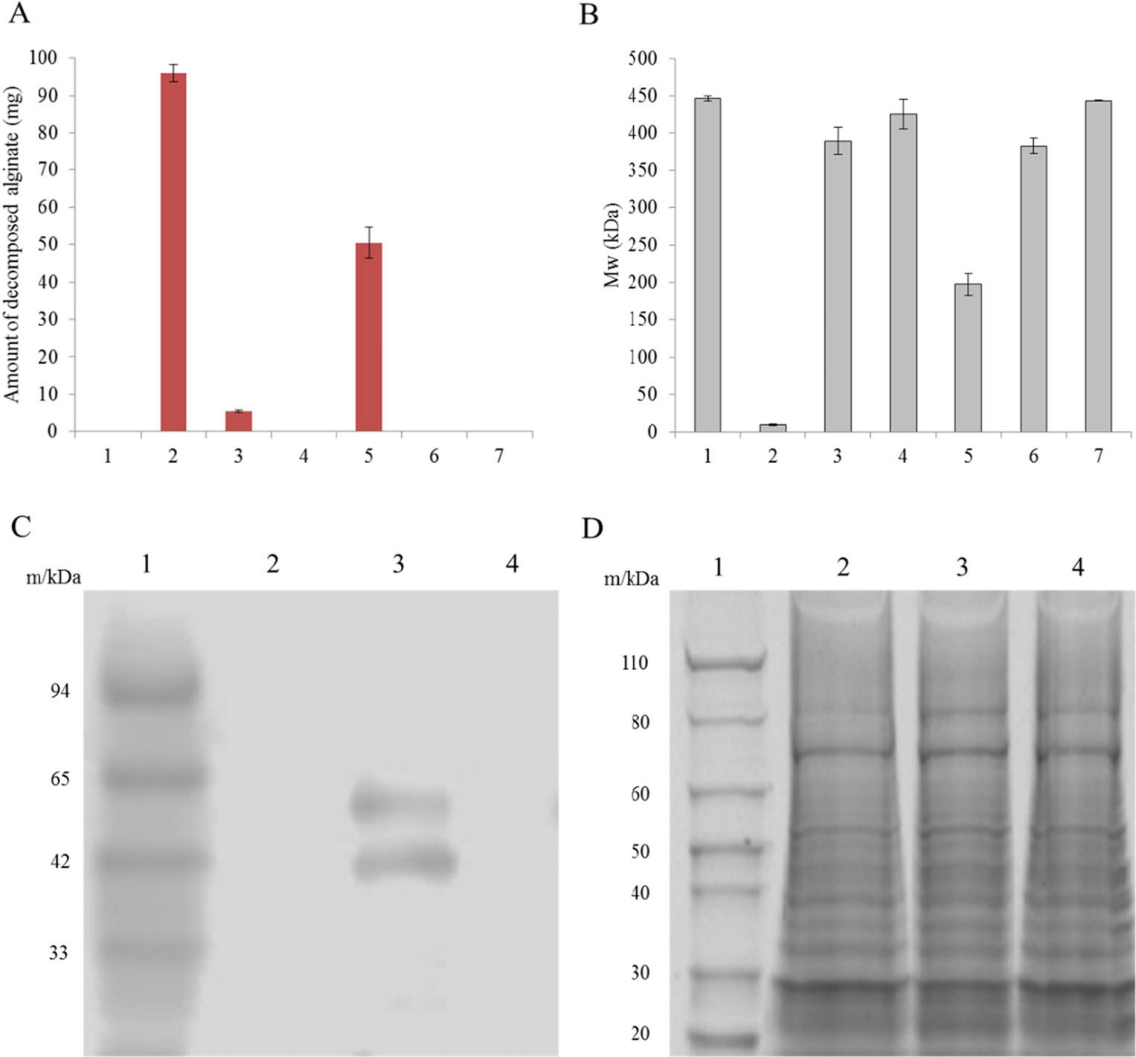
SP-dependent extracellular alginate decomposition. **a** Quantification of decomposed alginate in concentrated supernatant samples (with 100 mg sodium alginate added to 5-ml concentrated supernatant protein samples; protein concentration: 0.24 g/l). GPC analysis was carried out after shaking at 120 rpm and 37 °C for 20 h. *1*, JM109/pM01 (vector control); *2*, JM109/pM03 (expressing wild-type AlyB of *V. algivorus*); *3*, JM109/pM04 (SP deletion mutant); *4*, JM109/pM06 (PL7 deletion mutant); *5*, JM109/pM05 (CBM32 deletion mutant); *6*, JM109/pM07 (SP and CBM32 deletion mutant); *7*, JM109/pM02 (expressing wild-type AlyB of *V. splendidus*). **b** Average molecular weights after processing concentrated supernatant samples. Samples 1–7 are as described for panel **a**. **c** Western blot analysis of alginate in concentrated supernatants. *1*, XL-Western Marker SP-2170 (Aproscience, Tokushima, Japan); *2*, JM109/pM01 (vector control); *3*, JM109/pM03; *4*, JM109/pM04 (SP deletion mutant). **d** Sodium dodecyl sulphate polyacrylamide gel electrophoresis analysis alginate in concentrated supernatants. *Lane 1*, Novex Sharp Unstained Protein Standard (Thermo Fisher Scientific, Waltham, MA, USA); *lanes 2–4* are as described for panel **c**

We assessed the alginate depolymerization potential of the filtered and concentrated supernatants. The protein concentration of the supernatant was measured with the CBB protein assay, and the same amount of protein from each sample was incubated with alginate. The decrease in the amount of alginate and average molecular weights were evaluated by GPC (Fig. 5). We found that deletion of SP or PL7 reduced alginate-decomposing activity (Fig. 5a, b). A similar observation was made upon deletion of the CBM domain, although in this case half of the activity remained (Fig. 5a, b). A western blot analysis of the supernatants revealed a band of the same size as AlyB (57 kDa), which disappeared upon deletion of the SP domain (Fig. 5c).

### Artificial alginate assimilation by *E. coli* expressing alginate metabolism pathway genes of *V. algivorus*

**Fig. 6:**
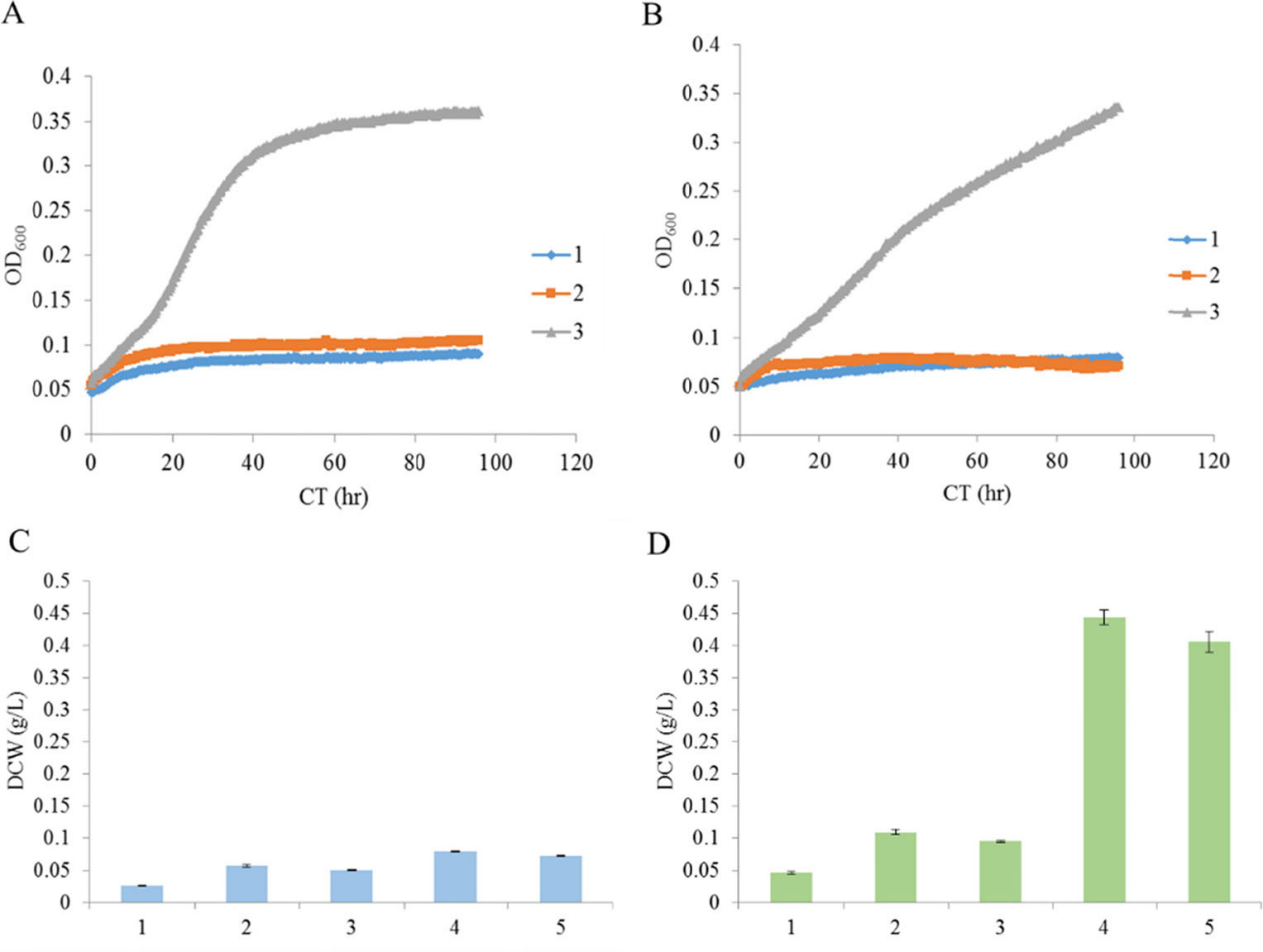
Results of test-tube cultivation on minimal medium. **a** Cell growth on minimal medium using alginate depolymerized with commercial alginate lyase as the sole carbon source. *1*, MG1655; *2*, D2964; *3*, D2978. **b** Cell growth on minimal medium using alginate depolymerized with AlyB (JM109/pM03) as the sole carbon source. *1*, MG1655; *2*, D2964; *3*, D2978. **c** MG1655 accumulation on M9 medium, presented as dry cell weight (DCW). *1*, M9 medium only (no carbon added); *2*, supernatant of JM109/pM03 cells (without alginate added); *3*, sodium alginate (without pre-processing); *4*, depolymerized alginate with AlyB-expressing cells (JM109/pM03); *5*, depolymerized alginate with commercial alginate lyase. **d** Accumulation of D2978 on M9 medium, presented as DCW. Samples *1–5* are as described for panel **c**

We searched the draft whole-genome sequence of SA2^T^ (Doi et al. 2016) for genes with sequences homologous to those encoding alginate utilization-related enzymes of *V. splendidus* 12B01 (Wargacki et al. 2012) using GENETYX v.10 software (GENETYX, Osaka, Japan). Seven such genes were identified (Table 3): *alyB*, *alyD*, *oalA*, *oalB*, *oalC*, *dehR*, and *toaA* were introduced into wild-type *E. coli* MG1655 cells, which were then cultured in M9 minimal medium with depolymerized alginate as the sole carbon source. Expression of the seven genes along with *alyB* enabled *E. coli* MG1655 cells to utilize the alginate that was depolymerized by AlyB activity (Fig. 6b, d).

**Table 3:** Candidate genes of *V. algivorus* encoding enzymes related to alginate degradation and metabolism

Assigned gene name	Annotated function of homologous genes of *V. splendidus* (Wargacki et al. 2012)	DNA similarity to homologous genes of *V. splendidus* (%)	GenBank/EMBL/DDBJ accession no.
*alyB*	Alginate lyase	69	LC175806
*alyD*	Alginate lyase	65	LC175802
*kdgN*	Porin	53	LC175810
*toaA*	Symporter	75	LC175801
*oalA*	Oligoalginate lyase	81	LC175805
*oalB*	Oligoalginate lyase	69	LC175803
*oalC*	Oligoalginate lyase	74	LC175804
*dehR*	DEHU reductase	81	LC175807

*DEHU* 4-deoxy-l-erythro-5-hexoseulose urinate

### Bioconversion of alginate to l-lysine

**Fig. 7:**
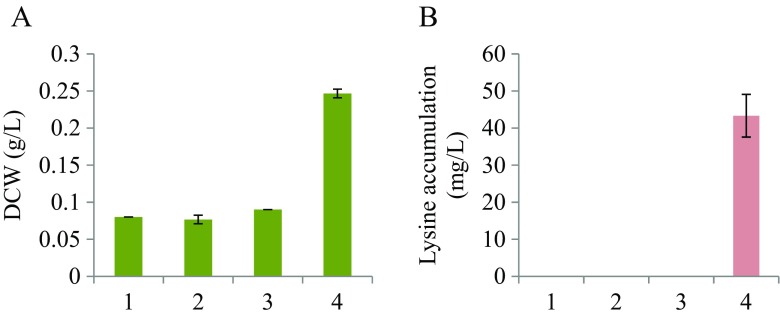
Results of test-tube cultivation for l-lysine bioconversion. **a** Dry cell weight (DCW) accumulation. **b** l-lysine accumulation. *1*, AJIK01 strain without depolymerized soluble alginate; *2*, D3000 strain without depolymerized soluble alginate; *3*, AJIK01 strain with alginate depolymerized by AlyB of *V. algivorus* expressed in *E. coli*; *4*, D3000 strain with alginate depolymerized by AlyB of *V. algivorus* expressed in *E. coli*

*E. coli* strain AJIK01 can utilize d-glucose and accumulate l-lysine (Doi et al. 2015). The introduction of the seven genes homologous to *V. algivorus* alginate metabolism genes (*alyB*, *alyD*, *oalA*, *oalB*, *oalC*, *dehR*, and *toaA*) into *E. coli* strain AJIK01 enabled the cells to utilize the alginate depolymerized by AlyB activity and accumulate l-lysine (Fig. 7a, b). There was no l-lysine accumulation in the absence of depolymerized alginate (Fig. 7b2) and without expression of all seven genes (Fig. 7b3).

## Discussion

Macroalgal utilization requires viscous polysaccharide decomposition. Brown macroalgae contain alginate, whereas red macroalgae contain carrageenan, xylan, and agarose (Chapman 1970); these are all high molecular weight polysaccharides that cannot pass through the cell membrane. It is therefore important to identify enzymes that can decompose these molecules. In this study, we used a simple double-layer screening procedure to identify AlyB, an extracellular polysaccharide-decomposing enzyme in *V. algivorus*, an alginate-assimilating strain. Like alginate, most polysaccharides are viscous; therefore, this double-layer screening procedure can be used to identify other polysaccharide-decomposing enzymes by adding a polysaccharide other than alginate to the upper layer gel.

Alginate degradation is important for its industrial applications (Wang da et al. 2014) and is necessary for microbial alginate assimilation. Many *Vibrio* species are presumed to have enzymes for extracellular alginate degradation; indeed, several *Vibrio* alginate lyases such as AlyVI from *Vibrio* sp. QY101 (Han et al. 2004) and OalA, OalB, OalC, AlyA, AlyB, AlyD, and AlyE from *V. splendidus* 12B01 (Jagtap et al. 2014; Badur et al. 2015) have been purified and characterized. *V. algivorus* AlyB exhibited alginate-depolymerizing activity when expressed in *E. coli* EPI300 and JM109 cells without requiring any purification or concentration.

We found that *V. algivorus* AlyB contained three predicted functional domains—i.e. SP, CBM32, and PL7. *V. splendidus* AlyB (Genbank accession no. EAP94922.1, Badur et al. 2015) also has these domains in the same order.

Deletion of PL7 resulted in the loss of alginate-depolymerizing activity, suggesting that the PL7 domain is the active centre of this enzyme, similar to the alginate lyase of *V. splendidus* (Badur et al. 2015).

Deleting the SP domain caused the alginate-decomposing activity to be lost in the supernatant and washed cells but not in the cell lysate. The GPC analysis confirmed that the lysate from cells expressing the SP deletion mutant showed a reduced peak corresponding to alginate (data not shown). However, a western blot analysis showed that the AlyB signal in the supernatant was undetectable upon deletion of SP. The most recent study of *V. splendidus* alginate lyase used mutant AlyB protein in which a His tag replaced the N-terminal SP for all the experiments and did not discuss the function of the SP (Badur et al. 2015). Our results show that wild-type AlyB of *V. splendidus* is not released into the supernatant irrespective of the presence of the SP; in contrast, in *V. algivorus*, the release of AlyB into the extracellular medium is dependent on this domain.

The CBM32 domain of *V. splendidus* AlyB is essential for its alginate lyase activity (Badur et al. 2015). In this study, deletion of the CBM32 domain of *V. algivorus* AlyB reduced the extracellular alginate depolymerization potential by 50%, whereas extra- and intracellular alginate-decomposing activity was not lost completely. These results suggest that the function of the CBM32 domain differs in the two species.

Alginate depolymerized by Aly enzymes has many uses, including as an ingredient of fish jelly food products (Sato et al. 2005), an agent that promotes plant growth (Yonemoto et al. 1995), and as an antioxidant (Falkeborg et al. 2014). However, these require alginate concentration and purification prior to depolymerization, which is unsuitable for large-scale applications due to the high cost. The fact that *V. algivorus* AlyB does not require purification makes it a good candidate for industrial production.

Uptake of alginate oligomer and its metabolism are required for alginate assimilation. Alginate-assimilating microbial species are presumed to have enzymes for degradation, uptake, and metabolism of this polysaccharide. Indeed, these have been reported for *V. splendidus* 12B01; expression of *toaA*, *toaB*, *eda*, *kdgK*, *oalA*, *oalB*, *oalC*, and *dehR* in *E. coli* strain ATCC8739 enabled the cells to utilize alginate depolymerized by alginate lyase derived from *Pseudoalteromonas* sp. SM0524 and expressed in another strain of *E. coli* (Wargacki et al. 2012). However, there are no reports describing the depolymerization of extracellular alginate for assimilation by alginate lyase of a specific *Vibrio* strain; the above-mentioned study used alginate lyase from a different species (*Pseudoalteromonas* sp. SM0524, not *V. splendidus*) for assimilation. In the present study, we expressed seven alginate utilization pathway genes derived from *V. algivorus* in *E. coli* MG1655; the cells began utilizing extracellular alginate depolymerized by the activity of *V. algivorus* AlyB as a sole carbon source, suggesting that AlyB has the same function in *V. algivorus*.

**Fig. 8:**
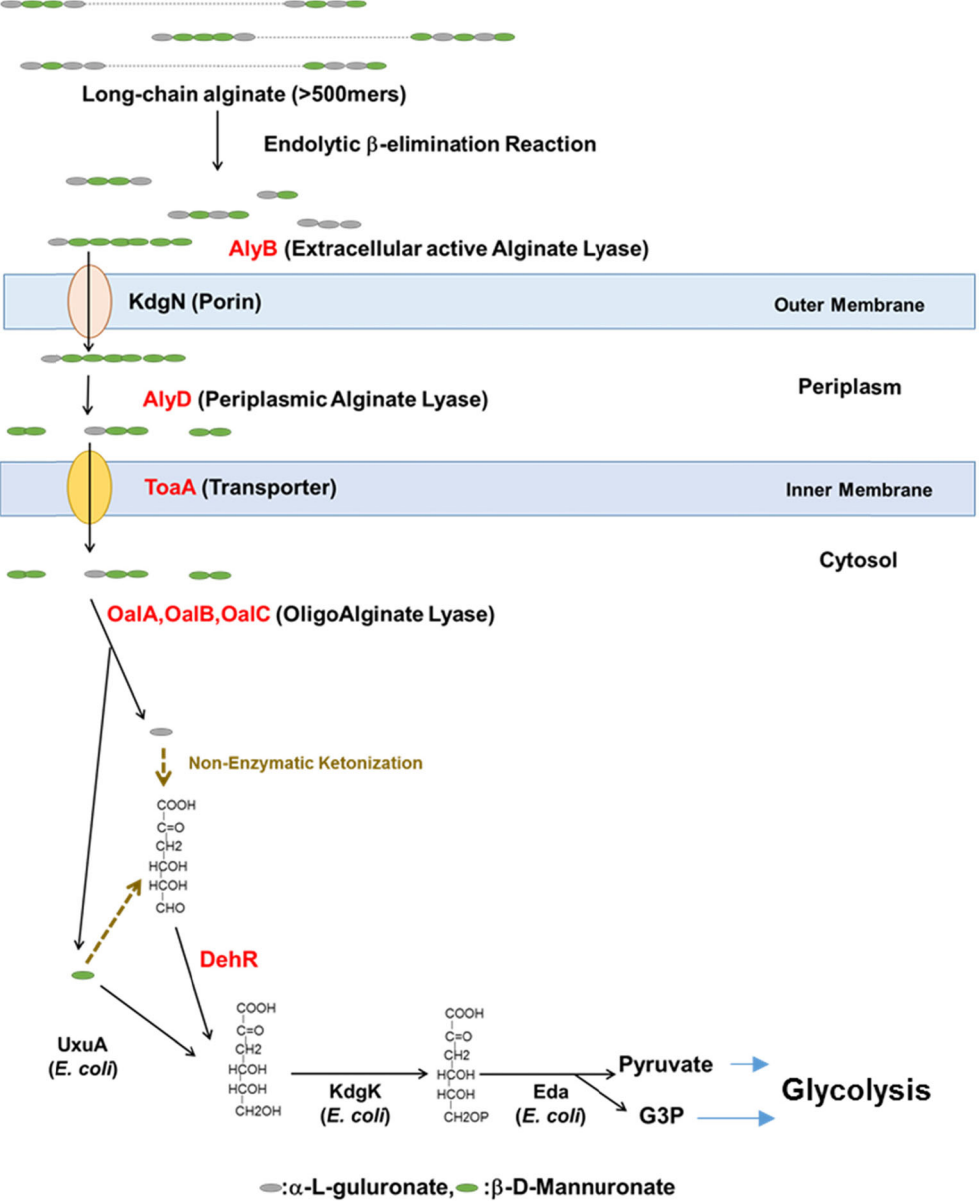
Model of alginate degradation and metabolism in *V. algivorus*. Proteins denoted in *red letter* are heterologously expressed in *E. coli* MG1655 and fulfilled their functions for alginate utilization in this study

ToaA expression in *E. coli* increased alginate utilization and cell growth, although the import of depolymerized soluble alginate into the cytosol by *V. algivorus* ToaA was a rate-limiting step. *E. coli* cells expressing ToaA solely were unable to grow using enzymatically decomposed extracellular alginate as the sole carbon source (data not shown). We therefore speculate that other enzymes involved in alginate metabolism (*alyB*, *alyD*, *oalA*, *oalB*, *oalC*, and *dehR*) also function in alginate utilization. Our results suggest that *V. algivorus* assimilates depolymerized alginate via a single uronic acid molecule (beta-d-mannuronate and 4-deoxy-l-erythro-5-hexoseulose urinate), similar to what has been predicted for *V. splendidus* (Wargacki et al. 2012) and *Sphingomonas* sp. strain A1 (Takeda et al. 2011). We propose a model of alginate degradation and metabolism in *V. algivorus* SA2^T^ based on our findings (Fig. 8).

In this study, we evaluated the potential for producing alginate derivatives using engineered *E. coli*. One possible derivative is l-lysine, a commodity chemical that is commercially produced by fermentation in the order of over 1,500,000 metric tons per year (Doi et al. 2014). There are no reports to date describing the bioconversion of alginate to l-lysine. We observed here that the alginate depolymerized by AlyB turned into cell biomass and l-lysine.

We have not introduced the previously reported technologies for improving L-lysine productivity of *E. coli* in this study. Concrete examples of such technologies are the introduction of feedback resistant L-lysine biosynthesis genes (Kojima et al. 1994), the introduction of heterologous *ddh* gene of *Corynebacterium glutamicum*, and the attenuation of the meso-α,ε-diaminopimelic acid synthesis pathway (Doi and Ueda 2009). In the future, L-lysine production from alginic acid can be further developed with introduction of these technologies.

Metabolically engineered *E. coli* fermentation can be used to produce other commodity chemicals such as l-glutamate (Nishio et al. 2013), l-tryptophan (Wang et al. 2013), l-phenylalanine (Báez-Viveros et al. 2007), lactic acid (Niu et al. 2014), and succinic acid (Zhu et al. 2013). Our results indicate that expressing *V. algivorus* genes in *E. coli* is a suitable alginate degradation and utilization system that can be used in combination with fermentation to produce commercially valuable chemicals.

A higher bioconversion efficiency is necessary for the industrialization of alginate fermentation. This requires optimizing the expression levels of alginate metabolism enzymes. A recent study reported that a high-throughput method for constructing recombinant variants known as recombinase-assisted genome engineering enhanced the efficiency of bioconversion of alginate into ethanol by recombinant *E. coli* (Santos et al. 2013). Other efficient high-throughput engineering technologies have also been reported for *E. coli* (Esvelt and Wang 2013; Ronda et al. 2016). These approaches can broaden the industrial applications of *V. algivorus* AlyB and other enzymes involved in alginate depolymerization and metabolism.

## Electronic supplementary material

ESM 1 (PDF 456 kb)
