# Supplementary material for: Identification of enzymes responsible for extracellular alginate depolymerization and alginate metabolism in Vibrio algivorus
Source: Appl Microbiol Biotechnol. 2016 Dec 3;101(4):1581–92. doi: 10.1007/s00253-016-8021-7 (PMC5266763; doi:10.1007/s00253-016-8021-7)
Supplement: Supplementary file 1 — (PDF 456 kb) [file 253_2016_8021_MOESM1_ESM.pdf]

Applied Microbiology and Biotechnology

Title

Identification of enzymes responsible for extracellular alginate depolymerization and alginate metabolism in *Vibrio algivorus*

Authors

Hidetaka Doi[1, 2] (Corresponding author), Yuriko Tokura[1], Yukiko Mori[1], Kenichi Mori[1], Yoko Asakura[1], Yoshihiro Usuda[1,3], Hiroo Fukuda[2], Akito Chinen[1]

Authors

[1]:Process Development Laboratories, Research Institute for Bioscience Products & Fine Chemicals, Ajinomoto Co., Inc.

1-1 Suzuki-cho, Kawasaki-ku, Kawasaki-shi, Kanagawa 210-8681, Japan

[2]:Department of Biological Sciences, Graduate School of Science, The University of Tokyo, 7-3-1 Hongo, Bunkyo-ku, Tokyo 113-0033, Japan

[3]: Frontier Research Laboratories, Institute for Innovation, Ajinomoto Co., Inc.

1-1 Suzuki-cho, Kawasaki-ku, Kawasaki-shi, Kanagawa 210-8681, Japan

Correspondence: Hidetaka Doi

e-mail: hidetaka_doi@ajinomoto.com

telephone: +81-44-245-5972

Table S1. Primers used in this study

Primer	Sequence	Description
EPI for	GGA TGT GCT GCA AGG CGA TTA AGT TGG	For sequencing pCC1FOS
EPI rev	CTC GTA TGT TGT GTG GAA TTG TGA GC	For sequencing pCC1FOS
L47	AGA TAT AAA ACC CTT ATA TAT TAA TAC GAT T	For amplifying pMW119- attR-cat-attL-P$_{14}$ linear fragment
L48	gtc gac tct aga gga tcc ccg ggt acc gag c	For amplifying pMW119- attR-cat-attL-P$_{14}$ linear fragment
N73	TAA GGG TTT TAT ATC TAT GAA ACA TAT TTT TCT AAA AAG CTT GCT AGC TTC	For amplifying *V. algivorus alyB* linear fragment
N74	atc ctc tag agt cga cTT ATT TAC CTG TGT ATG TAC CGT GCG ATT TTT CTA	For amplifying *V. algivorus alyB* linear fragment
N75	TAA GGG TTT TAT ATC TAT GTT AAA AAA ATT CCT CTG CAT GTC GGT TAT CCT	For amplifying *V. algivorus alyD* linear fragment
N76	atc ctc tag agt cga cCT ATT TTT TAT GCG ATG TTA ATT CTA ACT TAG AGA	For amplifying *V. algivorus alyD* linear fragment
N77	TAA GGG TTT TAT ATC TAT GAT GAC TAA ACC TGT TAT TGG TTT TAT CGG CCT	For amplifying *V. algivorus dehR* linear fragment
N78	atc ctc tag agt cga cTT ACT TTT TCA AAC CCA TGA AGT AAT CAA AGA TAA	For amplifying *V. algivorus dehR* linear fragment
O55	TAA GGG TTT TAT ATC Tat gac aga cca aaa atc tct tga tgc gat cag gaa	For amplifying *V. algivorus oalA* linear fragment
O56	ATC CTC TAG AGT CGA Ctt aca gcg taa taa caa cac ttt cac cat caa ca	For amplifying *V. algivorus oalA* linear fragment
O57	TAA GGG TTT TAT ATC Tat gaa aaa tga agt atc agc tgt att gct taa cac	For amplifying *V. algivorus oalB* linear fragment
O58	ATC CTC TAG AGT CGA Ctt att tca ctt gta ctt cta atg aat aga agc cat	For amplifying *V. algivorus oalB* linear fragment
O59	TAA GGG TTT TAT ATC Tat gaa tta cca acc att att aat gaa ttt tga aga	For amplifying *V. algivorus oalC* linear fragment
O60	ATC CTC TAG AGT CGA Ctt ata act gag ccg ttg ctc ccg tcc att ggt att	For amplifying *V. algivorus oalC* linear fragment
H69	TAA GGG TTT TAT ATC TAT GAA ACA AAT TAC TCT AAA AAC TTT ACT	For amplifying *Vibrio splendidus alyB* linear fragment
H70	atc ctc tag agt cga cTT ACT TTT TGT ATT GAT CGT GCG ATA CAT	For amplifying *Vibrio splendidus alyB* linear fragment
Q03	AAT GAA CAG TTG ACT GCT GCT GCA ACA CGC GGG CCT TCT TGT TTT GC	For amplifying pMW119-attR-*cat*-attL-P$_{14}$-*alyB*⊿CBM32 linear fragment
Q04	GCA GCA GTC AAC TGT TCA TTG AAC TCT	For amplifying pMW119-attR-*cat*-attL-P$_{14}$-*alyB*⊿CBM32 linear fragment
Q05	GGT ACA TAC ACA GGT AAA TAA	For amplifying pMW119-attR-*cat*-attL-P$_{14}$-*alyB*⊿PL7 linear fragment

Q06	ACC TGT GTA TGT ACC GTT TTG GCT TGG CTT ATT GCC	For amplifying pMW119-attR-*cat*-attL-P$_{14}$-*alyB*⊿PL7 linear fragment
Q07	aac agt tga ctg ctg cCA TAG ATA TAA AAC CCT TAT ATA TTA A	For amplifying pMW119-attR-*cat*-attL-P$_{14}$-*alyB*⊿SP⊿CBM32 linear fragment (used with Q10)
Q09	TAA GGG TTT TAT ATC TAT GGG CTG TTC ATC TAA TGG CGC CGA	For amplifying pMW119-attR-*cat*-attL-P$_{14}$-*alyB*⊿SP linear fragment
Q10	AGA TAT AAA ACC CTT ATA TAT TAA TA	For amplifying pMW119-attR-*cat*-attL-P$_{14}$-*alyB*⊿SP linear fragment
O01	ATA GCC GGG GCG GTC TTC CTG ATT GGT AGC TGG CTG CGT TAT GAC TAC GGT CTA GAC GCT CAA GTT AGT ATA AAA AAG CT	For introduction of ⊿*narI*::P$_{14}$-*dehR*
O04	CGG TAC GCT CCA GAT GTG TAT CAG ACG CGA GAA CGG GAA CAG CAG GAA TAT TAC TTT TTC AAA CCC ATG AAG TAA TCA AA	For introduction of ⊿*narI*::P$_{14}$-*dehR*
O20	gat ggt gac aga att aca gga gat acc gcc gat cca tca gga aac ctc taT CTA GAC GCT CAA GTT AGT ATA AAA AAG CT	For introduction of ⊿*narI*::P$_{14}$-*alyB*
O38	CCG TTA TTC CAG CCA TTA CCT TTG AAA CTG TAC TTC TCC Ctt att tac ctg tgt atg tac cgt gcg att ttt cta gtt ta	For introduction of ⊿*narI*::P$_{14}$-*alyB*
O32	tta ccg gtg tcg cta ttt tga aca tcc agc tct ggt att ccg caa aag caT CTA GAC GCT CAA GTT AGT ATA AAA AAG CT	For introduction of ⊿*narI*::P$_{14}$-*alyD*
O35	ggg tct gcg ttt acg cgg cca acg aaa ctg tga tca att ttg ata taa tcC TAT TTT TTA TGC GAT GTT AAT TCT AAC TT	For introduction of ⊿*narI*::P$_{14}$-*alyD*
O70	gtt acg ctt tcg ctt atg gcg cag atg ctg ttt atg cgg gcc agc TCT AGA CGC TCA AGT TAG TAT AAA AAA GCT GAA CG	For introduction of ⊿*narI*::P$_{14}$-*oalA*
O72	cac agt ata acc atc gcc tgg tgc tat cgg cat agc ttc gcc ttt tta cag cgt aat aac aac act ttc acc atc aac a	For introduction of ⊿*narI*::P$_{14}$-*oalA*
O65	CAG ACT TAT CTC ACT GAT CAC CCT GTA ACG TTC AGA GAG CGT CTT TCT AGA CGC TCA AGT TAG TAT AAA AAA GCT GAA CG	For introduction of ⊿*ybdN*::P$_{14}$-*oalB*
O67	cgc tga tag ttc ttc gat ttt gtg ggg cta aat gat aat gcc cga TTA TTT CAC TTG TAC TTC TAA TGA ATA GAA	For introduction of ⊿*ybdN*::P$_{14}$-*oalB*

	GCC AT	
O75	ATT CAC ATC CCG TGG TGC GTG CAG AAA TGC CCG TAC TGC GAT TTC TCT AGA CGC TCA AGT TAG TAT AAA AAA GCT GAA CG	For introduction of ⊿*yggW*::P_{14}-*oalC*
O77	aac agc ttc cca tgt tcc gtt atc tgc cag taa tcc gca cat tcg tta taa ctg agc cgt tgc tcc cgt cca ttg gta tt	For introduction of ⊿*yggW*::P_{14}-*oalC*
K01	TCG CGA CGG CAA CAT TTC GCT AAA GTC ACG CCC CTT CTT CAC CGG CAT GGG GAT TAT TTC TCT AGA CGC TCA AGT TAG TA	For construction of ⊿yegD::P_{tac6}-*toaA* fragment (crossover PCR) and its introduction
K02	GGC AGC AAC AAC AAT AGT ATC GAC AGT CAT agc tgt tTC CTG TGT GAA ATT GTT ATC CGC	For construction of ⊿yegD::P_{tac6}-*toaA* fragment (crossover PCR) and its introduction
K03	GCG GAT AAC AAT TTC ACA CAG GAa aca gct ATG ACT GTC GAT ACT ATT GTT GTT GCT GCC	For construction of ⊿yegD::P_{tac6}-*toaA* fragment (crossover PCR) and its introduction
K04	gca gca gaa aaa ctg gct cag gcg cag gca gat tta agc cgc tgc tga gcc att ttt caa TTA CGA TTC TGA AAC CGT TT	For construction of ⊿yegD::P_{tac6}-*toaA* fragment (crossover PCR) and its introduction

21

Table S2. Analytical conditions for GPC analysis

System:	SHIMADZU Prominence L C20A UFLC
Column:	Asahipak GS-520 HQ, 7.5 × 300 mm, 7 μm
Solvent:	100 mM KH_2PO_4
Flow rate:	0.6 ml/min
Injection volume:	60 μl
Detection wavelength:	200 nm

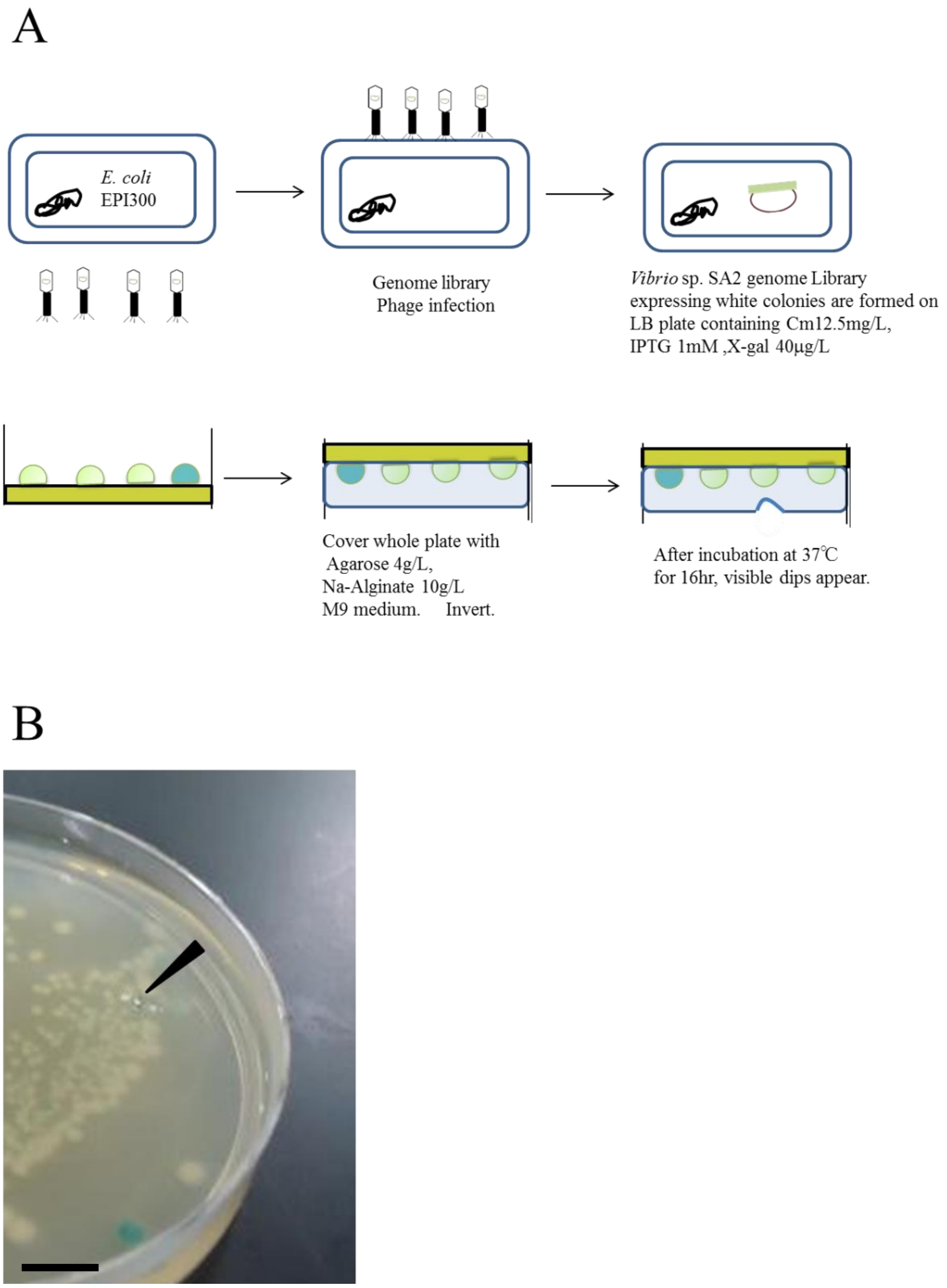

Figure S1. Screen for extracellular active alginate lyase. A) Schematic representation of the double-layer screening method, B) A visible dip (arrowhead) appeared above the colony expressing the candidate alginate lyase. Scale bar: 1cm
